# Risk of Falling and Associated Factors in Older Adults with a Previous History of Falls

**DOI:** 10.3390/ijerph17114085

**Published:** 2020-06-08

**Authors:** Begoña Pellicer-García, Isabel Antón-Solanas, Enrique Ramón-Arbués, Loreto García-Moyano, Vicente Gea-Caballero, Raúl Juárez-Vela

**Affiliations:** 1 Servicio Aragonés de Salud, Sector Alcañiz Atención Primaria, Centro de Salud Andorra Calle Huesca s/n, 44500 Andorra, Teruel, Spain; bpellicerg@salud.aragon.es; 2 Faculty of Health Sciences, Universidad San Jorge, Campus Universitario, Villanueva de Gállego, Autovía A-23 Zaragoza-Huesca Km. 299, 50830 Villanueva de Gállego, Zaragoza, Spain; eramon@usj.es; 3 Servicio Aragonés de Salud, Hospital General San Jorge, Av. Martínez de Velasco 36, 22004 Huesca, Spain; loretongarcia@hotmail.com; 4 Nursing School La Fe, adscript center of University of Valencia, Research Group GREIACC, Health Research Institute La Fe, Valencia (Spain), Pabellón Docente, Torre H, Avinguda de Fernando Abril Martorell 106, 46026 Valencia, Spain; gea_vic@gva.es; 5 School of Nursing, University of La Rioja, Research Group PBM Idi-Paz, C/Duquesa de la Victoria 88, 26004 Logroño, La Rioja, Spain; raul.juarez@unirioja.es

**Keywords:** accidental falls, aged, gait, postural balance, risk factors

## Abstract

Falls in the elderly are one of the main geriatric syndromes and a clear indicator of fragility in the older adult population. This has serious consequences, leading to an increase in disability, institutionalization and death. The purpose of this cross-sectional study was to analyze the prevalence of risk of falling and associated factors in a population of 213 non-institutionalised, able older adults with a history of falling in the previous year. We used the following assessment tools: Questionnaire of the WHO for the study of falls in the elderly, Geriatric Depression Scale and Tinetti’s Gait and Balance Assessment Tool. Age, using ambulatory assistive devices, polymedication, hospital admission following a fall and depression were significantly associated with risk of falling. In order to prevent fall reoccurrence, community-based fall prevention programs should be implemented.

## 1. Introduction

Falls in the elderly are one of the main geriatric syndromes and a clear indicator of fragility in the older population [1]. Out of older adults in the sample, 30–45% who live in the community will experience at least one fall per year [2]. This has serious consequences, leading to an increase in disability, institutionalization and death [1]. Worldwide, falls are the second cause of mortality, with a rate of 424.000 deaths annually [3].

Given the multi-causality of falls in the older population, both first time and recurrent, it is important to implement a multifactorial assessment of risk of falling. The risk factors for falls are multiple and interrelated, and include having a history of previous falls, gait alterations, osteoporosis, loss of functional capacity, fear of future falls, visual alterations, impaired cognitive capacity, urinary incontinence, cardiovascular problems, medication intake, fatigue and the environment [4,5,6].

The aim of this study is to analyze the prevalence of risk of falling and associated factors in a population of non-institutionalized, able older population with a history of falling in the previous year in Spain.

## 2. Materials and Methods

A cross-sectional study was performed involving a random sample of 213 non-institutionalized older people in the city of Zaragoza (Spain), between May-July 2015.

Participants were randomly selected from among a total population of 16,000 users of two social centers for older adults in Zaragoza city center. We recruited older adults who voluntarily agreed to take part in the study and met the inclusion criteria, namely age ≥ 65, non-institutionalized, not cognitively impaired (as determined by the Pfeiffer Short Portable Mental Questionnaire (PSMSQ) in its Spanish version) [7] independent individuals who had had at least one fall in the last 12 months prior to the start of the study. The following were exclusion criteria for participation: (1) Being under 65, (2) not having experienced at least one fall in the last 12 months, (3) being unable or refusing to be interviewed, and (4) scoring 3 or more in the Short Portable Mental State Questionnaire (SPMSQ). The final sample consisted of 213 participants. The sample size was calculated with Statistical Package for the Social Sciences SPSS© (version 21 for Windows, IBM Corp., Armonk, NY, USA) for a 30% prevalence of falls in the chosen population, as determined in the literature [8,9], a confidence level (1- α) of 95%, an accuracy (d) of ±0.5, a variance (S2) of 13.72 and an estimated 5% drop out rate.

Face-to-face, individual interviews were arranged by telephone and took place in the two social centers. All the assessment tools had been previously validated for use in the Spanish speaking population. The questionnaire of the WHO for the study of falls in the elderly [10] was designed to assess the circumstances of the fall using the following variables: Use of ambulatory assistive devices, urinary incontinence, fear of falling, recurrent falls (≥ 2 in the previous year), consequences post-fall, use of health services and hospital admission following a fall.

Depression was assessed using Yesavage’s Geriatric Depression Scale (GDS) in its Spanish version [11,12]. This tool measures the symptoms of depression among older people with a maximum score of 15. A score ≤ 4 indicates absence of depression, while a score of five or higher is suggestive of clinical depression.

The dependent variable, risk of falling, was measured by Tinetti’s Gait and Balance Assessment Tool [13,14]. This test has two sections: The gait scale (12 points) and the balance scale (16 points). The higher the final score, the better the patient’s functionality and the lower the risk of experiencing a fall. Scores under 24 points indicate risk of falling. A qualified nurse with experience in care of the elderly conducted this assessment.

Data codification, processing and analysis were completed using the statistical software SPSS© (21 version for Windows). The main estimates were presented with a level of statistical significance p ≤ 0.05. We performed a univariate statistical analysis and a bivariate analysis (Chi-square and independent t-test). Binary logistic regression was applied to examine independent variables significantly associated with risk of falling. Specifically, the Enter method (including in the model only those variables which were statistically significant after bivariant analysis) was applied to examine independent variables significantly associated with risk of falling.

The study proposal was reviewed and approved by the Clinical Research Ethics Committee of Aragón (IRB Ref: PI13/00121) prior to the commencement of this study. All subjects gave written informed consent before being interviewed.

## 3. Results

A total of 213 participants (169 women and 44 men) took part in this study. Mean age was 78 (SD ± 6.999). Approximately a quarter of our participants used assistive devices for ambulation and had a previous diagnosis of depression, whereas more than 40% were incontinent of urine or lived alone. Our 12-month prevalence of recurrent falls was almost 75%, with over 60% of our population expressing a fear of falling. Over three quarters of our participants said that they had experienced negative consequences post-fall, with just over 50% seeking medical assistance.

After univariate analysis, we found that approximately one third (31.9%) of the participants were at risk of falling. The effect of the independent variables on the dependent variable (risk of falling) was analyzed through X2 (95% CI); statistically significant relationships were found between risk of falling and using assistive devices for ambulation, taking ≥ 4 medications daily, being admitted to hospital after a fall and having a diagnosis of depression. A significant association with the variable age was also found through an independent t-test (Table 1).

Binary logistic regression analysis was applied to determine the independent variables’ effect on the participants’ risk of falling. We discovered that a Tinetti score ≤ 24 was a risk factor for age (OR:1.092, p ≤ 0.001), using assistive devices for ambulation (OR:20.187, p ≤ 0.001), taking ≥ 4 drugs daily (OR:4.261, p = 0.013), hospital admission post-fall (OR:5.347, p = 0.007) and depression (OR:11.240, p ≤ 0.001) (Table 2).

An analysis of prognostic probability revealed that a woman aged 84, who takes ≥ 4 medicines daily, lives alone, uses assistive devices for ambulation, is incontinent of urine, has a diagnosis of depression, has a fear of future falls and was admitted to hospital after a previous fall, will fall again with a 99.5% probability.

## 4. Discussion

We observed a high prevalence of risk of falling in our population. Using assistive devices for ambulation, including canes, one or two crutches, walkers and wheelchairs, was strongly associated with the risks of falling, suggesting that both gait and balance may be impaired in older adults who use technical aids for mobility. This may seem paradoxical, and as such, devices have been designed to increase the user’s base of support and improve their balance. However, factors associated with the use of these devices, such as self-prescription, lack of training, inadequate height, poor maintenance and preexisting patient conditions or characteristics, including a lack of strength and endurance; visual, cognitive or vestibular function impairment; and environmental demands, have been identified in the literature [15]. Interestingly, a more recent study [16] showed that elderly users of ambulatory assistive devices fall mainly when they are not using them, suggesting that education interventions should aim, not only to provide training in the use of these devices, but also encourage older adults to use them during the activities of daily living. These variables are important and should be considered when caring for older adults in need of mobility aids. However, other factors, such as frailty should be considered when investigating the relationship between assistive devices and falls in the elderly [17].

Depression was also strongly associated with risk of falling. This is in agreement with previous studies [18,19,20], which demonstrated that risk of falling was approximately twice as high in older people with depression. Choi et al. [21] identified a higher prevalence of falls in non-institutionalized older adults whose mean age was 73.2, and in those who had depression, were visually impaired and were incontinent of urine. In contrast to the previous study, our participants had had at least one fall in the 12 months prior to the start of the research. However, both depression and age (mean age in our study was slightly higher) were also associated with risk of falling. No significant associations were found between urinary incontinence and risk of falling.

The fear of falling is common in this population, with a prevalence of 25–50%, and is associated with female sex, previous history of falls, gait alteration and symptoms of depression [22]. A higher prevalence of fear of falling was found in our study. This may be due to the fact that our participants had experienced at least one fall prior to taking part in the study, and therefore, their fear of falling again was higher [23].

Worth mentioning is the fact that almost three quarters of our participants were recurrent fallers. That is, they had had at least two falls in the 12 months previous to the study. A similar prevalence of recurrent falls was reported by Abreu et al [24] in a study involving community-dwelling older adults. We did not find a significant association between recurrent falls and risk of falling. However, older adults who fall are at risk of experiencing a repeated fall [25] and should be targeted in fall prevention programs.

Several studies confirm that polymedication increases the risk of falling, being the number of drugs taken more significant than the type of drugs [18,19,20,21,22,23]. Our results suggest that taking ≥ 4 medicines daily is significantly associated with risk of falling.

Only a small percentage of our participants needed hospital admission following a fall. However, we found an association between hospital admission after a fall and risk of falling. In the States alone, fall-related injuries lead to approximately 800,000 hospitalizations per year [26], costing more than $50 billion [27]. At individual level, the human cost of falling includes higher risk of death, pain, injury, distress and loss of confidence, and frequently derives in loss of independence and institutionalization [28].

Our findings indicate a high risk of recurrent falls in a population of community dwelling older people. Interventions, including information and advice on risks and fall prevention, the provision and training in the use of walking aids and promotion of global muscle strengthening exercises [29,30] should be implemented to reduce the risk of falling.

This study has some limitations that need to be acknowledged. First, the retrospective design of the study may have resulted memory bias. Although, it is unlikely that the risk of falling in our population has changed significantly in the past five years, we would like to acknowledge that our data is no longer considered recent.

## 5. Conclusions

The present study provides interesting and clinically relevant data, indicating that age, using ambulatory assistive devices, taking ≥ 4 medicines daily, being admitted to hospital after a fall, and having a diagnosis of depression, are strongly associated to risk of falling in non-institutionalized, able older adults, with a history of falling in the previous year.

It is important to reduce the risk of falling in this population, in order to prevent recurrent falls and associated consequences. Based on our findings and those already available in the literature, we recommend that future investigations in this area examine the risk factors for falling in this population. In addition, community-based fall prevention programs should be implemented allowing us to prevent first-time and recurrent falls and improve existing clinical guides for the prevention of falls in the older population. From a clinical point of view, risk of falling must be assessed frequently in older people as it is a good detector of fragility and contributes to avoid and prevent future falls and delay dependency and disability.

## Figures and Tables

**Table 1:** Univariate and bivariate analysis for risk of falls (Chi-square and independent t-test).

Variables	Total Participants	≥25 ^a^	≤24 ^b^	Z/t	p
	N = 213 (100%)	Tinetti	Tinetti		(<0.05)
		N = 145 (68.1%)	n= 68 (31.9%)		
Sex:					
Men	44 (20.7)	28 (19.3)	16 (23.5)	0.709	0.478
Women	169 (79.3)	117 (80.7)	52 (76.5)		
Age:					
Mean	78	75.3	81.5	−6.615	<0.001
(± SD)	± 6.999	±6.512	±6.075		
Assistive devices for ambulation:					
Yes	50 (23.5)	7 (4.8)	43 (63.2)	9.376	<0.001
No	163 (76.5)	138 (95.2)	25 (36.8)		
Living alone:					
Yes	94 (44.1)	67 (46.2)	27 (39.7)	−0.891	0.373
No	119 (55.9)	78 (53.8)	41 (60.3)		
Urinary incontinence:					
Yes	89 (41.8)	56 (38.6)	33 (48.5)	1.367	0.172
No	124 (58.2)	89 (61.4)	35 (51.5)		
Drug intake:					
≤3	73 (34.3)	63 (43.4)	10 (14.7)	−4.120	<0.001
≥4	140 (65.7)	82 (56.6)	58 (85.3)		
Fear of falling:					
Yes	129 (60.6)	85 (58.6)	44 (64.7)	0.847	0.397
No	84 (39.4)	60 (41.4)	24 (35.3)		
Recurrent falls:					
Yes	159 (74.6)	104 (71.7)	55 (80.9)	1.432	0.152
No	54 (25.4)	41 (28.3)	13 (19.1)		
Consequences post-fall:					
Yes	178 (83.6)	124 (85.5)	54 (79.4)	−1.121	0.262
No	35 (16.4)	21 (14.5)	14 (20.6)		
Use of health services:					
Yes	110 (51.6)	73 (50.3)	37 (54.4)	0.554	0.58
No	103 (48.4)	72 (49.7)	31 (45.6)		
Hospital admission following a fall:					
Yes	23 (10.8)	10 (6.9)	13 (19.1)	2.679	0.007
No	190(89.2)	135 (93.1)	55 (80.9)		
Depression/Yesavage:					
≥5 ^c^	60 (28.2)	19 (13.1)	41 (60.3)	7.138	<0.001
≤4 ^d^	153 (71.8)	126 (86.9)	27 (39.7)		

CI: 95%. ^a^ No risk of falling. ^b^ At risk of falling. ^c^ With depression. ^d^ No depression.

**Table 2:** Binary logistic regression analysis of the independent variables effect on risk of falling.

	B	Sig.	Exp(B)	C.I. 95% for EXP(B)	
				Inferior	Superior
AGE	0.088	0.019	1.092	1.015	1.176
ASSISTIVE DEVICES FOR AMBULATION	3.005	0.000	20.187	6.542	62.287
≥ 4 MEDICINES DAILY	1.450	0.013	4.261	1.351	13.438
HOSPITAL ADMISSION	1.677	0.015	5.347	1.388	20.595
DEPRESSION	2.419	0.000	11.240	4.169	30.302
Constant	−10.554	0.000	0.000		

Exp(B): Odds ratio. Sig.: Level of significance. C.I.: Confidence interval.
